# Gut–liver microphysiological systems revealed potential crosstalk mechanism modulating drug metabolism

**DOI:** 10.1093/pnasnexus/pgae070

**Published:** 2024-02-09

**Authors:** Dhimas Agung Kurniawan, Sylvia Leo, Mutsumi Inamatsu, Sohei Funaoka, Taichi Aihara, Mizuno Aiko, Inoue Rei, Takeshi Sakura, Hiroshi Arakawa, Yukio Kato, Tomoaki Matsugi, Katsuhiro Esashika, Nobuaki Shiraki, Shoen Kume, Kenta Shinha, Hiroshi Kimura, Masaki Nishikawa, Yasuyuki Sakai

**Affiliations:** Department of Chemical System Engineering, Graduate School of Engineering, University of Tokyo, Tokyo 113-8656, Japan; School of Life Science and Technology, Tokyo Institute of Technology, Kanagawa 226-8501, Japan; PhoenixBio Co. Ltd., Higashi-Hiroshima, Hiroshima 739-0046, Japan; Sumitomo Bakelite Co. Ltd., Tokyo 140-0002, Japan; Sumitomo Bakelite Co. Ltd., Tokyo 140-0002, Japan; Sumitomo Bakelite Co. Ltd., Tokyo 140-0002, Japan; Sumitomo Bakelite Co. Ltd., Tokyo 140-0002, Japan; Sumitomo Bakelite Co. Ltd., Tokyo 140-0002, Japan; Faculty of Pharmacy Institute of Medical, Pharmaceutical and Health Science, Kanazawa University, Kanazawa 920-1192, Japan; Faculty of Pharmacy Institute of Medical, Pharmaceutical and Health Science, Kanazawa University, Kanazawa 920-1192, Japan; Mitsui Chemicals Inc., Tokyo 104-0028, Japan; Mitsui Chemicals Inc., Tokyo 104-0028, Japan; School of Life Science and Technology, Tokyo Institute of Technology, Kanagawa 226-8501, Japan; School of Life Science and Technology, Tokyo Institute of Technology, Kanagawa 226-8501, Japan; Micro/Nano Technology Center, Tokai University, Kanagawa 259-1292, Japan; Micro/Nano Technology Center, Tokai University, Kanagawa 259-1292, Japan; Department of Chemical System Engineering, Graduate School of Engineering, University of Tokyo, Tokyo 113-8656, Japan; Department of Chemical System Engineering, Graduate School of Engineering, University of Tokyo, Tokyo 113-8656, Japan

**Keywords:** liver, small intestine, microphysiological system (MPS), organ crosstalk, drug metabolism

## Abstract

The small intestine and liver play important role in determining oral drug's fate. Both organs are also interconnected through enterohepatic circulation, which imply there are crosstalk through circulating factors such as signaling molecules or metabolites that may affect drug metabolism. Coculture of hepatocytes and intestinal cells have shown to increase hepatic drug metabolism, yet its crosstalk mechanism is still unclear. In this study, we aim to elucidate such crosstalk by coculturing primary human hepatocytes harvested from chimeric mouse (PXB-cells) and iPSc-derived intestinal cells in a microphysiological systems (MPS). Perfusion and direct oxygenation from the MPS were chosen and confirmed to be suitable features that enhanced PXB-cells albumin secretion, cytochrome P450 (CYP) enzymes activity while also maintaining barrier integrity of iPSc-derived intestine cells. Results from RNA-sequencing showed significant upregulation in gene ontology terms related to fatty acids metabolism in PXB-cells. One of such fatty acids, arachidonic acid, enhanced several CYP enzyme activity in similar manner as coculture. From the current evidences, it is speculated that the release of bile acids from PXB-cells acted as stimuli for iPSc-derived intestine cells to release lipoprotein which was ultimately taken by PXB-cells and enhanced CYP activity.

**Significance Statement**

As the absorption and metabolism site for oral drugs, gut–liver axis is important point of study in drug development. Understanding how gut affects liver drug metabolism activity or vice versa is challenging to do using in vivo approaches. Multiorgan microphysiological system (MPS) is a promising in vitro approach which allows focused study on specific organ combinations. This study revealed potential crosstalk mechanism between gut and liver which may involve bile acids, intestinal lipoprotein, and arachidonic acid that affects hepatic drug metabolism. At the same time, this study also demonstrated the importance of appropriate MPS features (perfusion, direct oxygenation) to effectively maintain cell functionality which ultimately allows studying novel multiorgan crosstalk.

## Introduction

The small intestine and liver are important organs which determine oral drug's fate. Following administration, drugs are absorbed in the small intestine where intestinal epithelial cells uptake the drugs and efflux them into the portal vein to be carried into the liver (1). Majority of drugs are metabolized by drug metabolizing enzyme cytochrome P450 (CYP) in the liver, forming metabolites which then distributed throughout the body by systemic circulation. Some drugs and endogenous molecules could also be reabsorbed to the small intestine after elimination from the liver through biliary excretion in a process commonly known as enterohepatic circulation (2). Such interaction between liver and intestine affects the efficacy and toxicity, which are crucial factors in the design of new drugs.

To predict efficacy and toxicity for new drugs, in vitro models have been widely used. In the recent years, there is growing attention in using microphysiological systems (MPS) as drug development tool (3). MPS allows advanced cell culture with various features such as perfusion, 3D structures, multicellular environment, and many other that could be tailored to specific applications (4, 5). Several studies have demonstrated usage of multiorgan MPS using liver and small intestine cells as drug development tools. Marin et al. (6) used two-organ compartment MPS, coculturing Caco-2 cell line in the small intestine compartment and organoid from HepaRG cells in the liver compartment under perfusion flow to study pharmacokinetics of acetaminophen. Their results showed that kinetics profile of acetaminophen was better achieved in the MPS with both liver and intestine cells compared to liver-only group. Another study by Arakawa et al. (7) using two-organ MPS comprised of similar cells evaluated the kinetics of triazolam metabolism. They found that clearance of triazolam metabolite in the coculture using two-organ MPS was higher than monoculture system. Previous study from our group (8) also observed similar findings using normal cells instead of cell lines. Intestinal cells derived from human-induced pluripotent stem cells (iPSc) and fresh human hepatocytes isolated from chimeric mice were used in a two-organ MPS. Albumin production and CYP activity of the hepatocytes were enhanced due to coculture with the iPSc-derived intestinal cells. Different protein expression in the cocultured cells compared to the monoculture was also observed. These studies confirmed that drug metabolism can be better recapitulated using multiorgan MPS compared to conventional monoculture. It can be inferred that organ crosstalk between the gut and liver is enhancing hepatic drug metabolism, although the crosstalk mechanism is still unclear.

Here, we aim to elucidate the mechanism of such crosstalk considering the following points: (i) both hepatocytes and intestinal cells must be maintained at their normal functions for them to exert normal crosstalk and (ii) the culture system must allow exchange of crosstalk factors between the two distant cells. For the first point, we chose to use PXB-cells and iPSc-derived intestine cells. PXB-cells are human hepatocytes freshly isolated from chimeric mice. It has been reported to have higher CYP enzyme activity compared to cryopreserved human hepatocytes, comparable CYP mRNA expression with adult primary human hepatocytes and stable albumin production expression for at least 21 days (9, 10). iPSc-derived intestinal cells are advantageous due to its human-derived nature and consistent supply. It reportedly exhibits higher transporter and CYP3A4 expression than the Caco-2 cell line, while maintaining comparable transporter function and expressing several CYP mRNAs similar to human adult intestine cells (11). We also added direct oxygenation by using oxygen permeable membrane as the well bottom material, which should help maintaining normal functions, especially for hepatocytes because of their high oxygen consumption rate (12). For the second point, a medium perfusion system between the two cells would be ideal to support bulk transport of common interorgan signaling factors such as nutrients, hormones, cytokines, chemokines, and extracellular vesicles (13). To fulfill those considerations, we used a two-organ MPS with on-chip perfusion system using kinetic pump in the microchannel (14). Here, we demonstrated that MPS with appropriate features can facilitate study on crosstalk mechanism for specific organ combinations, which may be challenging to do by using in vivo approaches. Specifically, our results suggest the involvement of bile acids, intestinal lipoprotein, and arachidonic acid (ARA) inside the gut–liver crosstalk which enhanced hepatic CYP activity.

## Methods

### Cell preparation

PXB-cells obtained from PhoenixBio Co. Ltd. (Hiroshima, Japan) were used as the liver model. Prior to seeding, MPS devices were treated with oxygen plasma to increase hydrophilicity. Then, culture wells were coated with 10% solution of Collagen type I-P (Nitta Gelatin, Japan) at 4 °C overnight to promote cell attachment. PXB-cells were seeded at density of 210,000 cells/cm^2^ with 1.0 mL medium per well and precultured for 4 days with medium exchange every 2 days before the coculture. Preculture medium composed of Dulbecco’s Modified Eagle’s Medium (DMEM) supplemented with 10% Fetal Bovine Serum (FBS), 44 mM NaHCO_3_, 20 mM HEPES, 1% penicillin–streptomycin, 15 μg/mL l-proline, 0.25 μg/mL insulin, 50 nM dexamethasone, 5 ng/mL h-EGF, and 0.1 mM l-ascorbic acid 2-phosphate.

iPSc-derived intestine cells were used as the small intestine model. The cells were obtained through maturation of iPSc-derived intestinal progenitor cells where the detailed information on differentiation and maturation methods has been reported elsewhere (11). Cryopreserved progenitor cells were thawed and plated into ad-MED Collagen Vitrigel culture inserts (Kanto Chemical, Japan). Rock inhibitor 10 μM Y-27632 were added to the culture medium during thawing until the first 2 days. The cells were cultured until maturation for 8 days with medium exchange every 2 days. Culture medium used for the thawing and maturation of iPSc-derived intestine cells consists of DMEM supplemented with 10% Knockout Serum Replacement, 1% penicillin–streptomycin, 2 mM l-glutamine, 1% MEM NEAA, 0.1 mM 2-mercaptoethanol, 10 μM *N*-[*N*-(3,5-difluorophenacetyl)-l-alanyl]-*S*-phenylglycine t-butyl ester (DAPT, Fujifilm Wako, Japan), and 5 μM 6-bromoindirubin-3′-oxime (Bio, Fujifilm Wako).

### Coculture in the MPS device

**Fig. 1.**
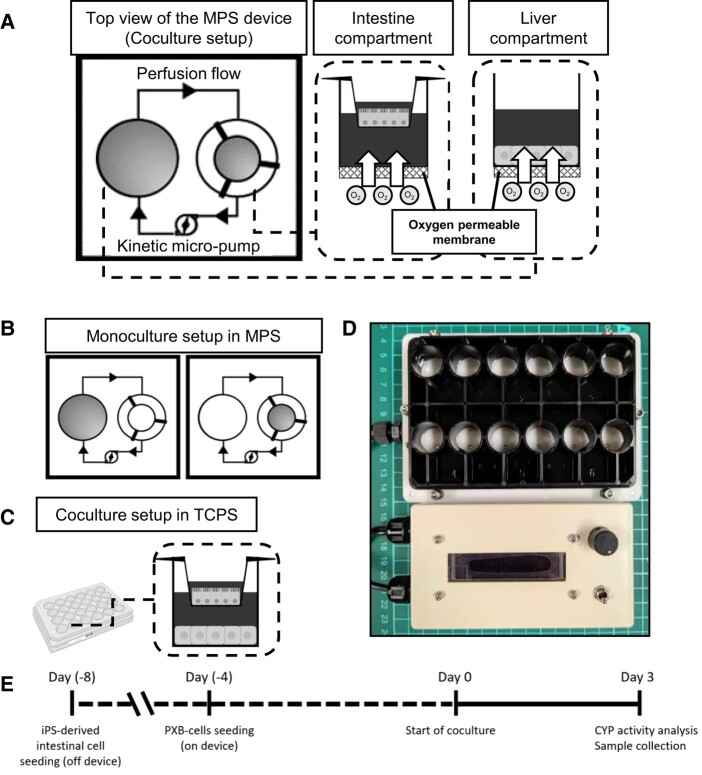
A) Diagram of the MPS-based in vitro liver–intestine coculture system. One unit consists of two-organ compartments (total 6 units per plate). Microstirrer pump placed in the microchannel connecting the two compartments provides perfusion to the system. The oxygen permeable membrane made of PMP was used as a base for the MPS device to provide increased oxygen supply. B) Monoculture setup of PXB and iPSc-derived intestinal cells in the MPS. C) Coculture setup in TCPS plate. D) Photograph of the MPS plate with its controller. E) Experiment timeline of preparation and coculture.

The MPS provided perfusion and direct oxygenation as its main features (Fig. 1). Perfusion was achieved by using kinetic pump inside the microchannel which is operated magnetically by external motor placed in the base of the device. Direct oxygenation was achieved by using oxygen permeable membrane as the well bottom material. The oxygen permeable membrane was made from 4-polymethy-1-pentene (PMP), a proprietary material from Mitsui Chemicals, Inc., which has shown superior properties compared to PDMS, especially in terms of adsorption of drug molecules (15). As control condition, conventional cell culture method (no perfusion and direct oxygenation) in tissue culture polystyrene (TCPS) plate was used. After seeding of PXB-cells in the MPS, culture inserts with mature iPSc-derived intestine cells were placed onto the complementary compartment to begin the coculture. The perfusion rate was adjusted by setting the kinetic pump rotation at 5,500 rpm, which corresponds to 35 μL/min (14). Coculture was performed for 3 days without medium change. The coculture medium consisted of DMEM supplemented with 2% FBS, 44 mM NaHCO_3_, 20 mM HEPES, 1% penicillin–-streptomycin, 15 μg/mL l-proline, 0.25 μg/mL insulin, 50 nM dexamethasone, 5 ng/mL h-EGF, and 0.1 mM l-ascorbic acid 2-phosphate.

### ARA treatment in PXB-cell culture

To study the effect of ARA compared to cocultured PXB-cells, 100 μM ARA (MP Biomedicals, USA) was added into the culture medium of monocultured PXB-cells. This concentration was selected as it is within the reported range of ARA plasma concentration (16). The duration of experiment follows the coculture period mentioned above.

### Albumin secretion measurement

Albumin concentration in the culture medium aliquots taken at the end of coculture was measured by sandwich enzyme-linked immunosorbent assay. Microplates were incubated with goat antihuman albumin antibody at 4 °C overnight, washed 3 times with 0.1% Tween-20/PBS, and blocked with blocking buffer containing 1% bovine gelatin for 2 h at room temperature. Samples were diluted accordingly, added into the microplates and incubated for 2 h at room temperature. Following another washing step, the microplates were incubated with HRP-conjugated goat antihuman albumin detection antibody for 2 h at room temperature. After final washing step, color development was performed, 3,3′,5,5′-tetramethylbenzidine substrate solution was added and incubated for 3 to 5 min in the dark. Color development was stopped by adding 1 M sulfuric acid. The absorbance was read using iMark microplate reader (Bio-Rad, USA) at 450 and 570 nm.

### Transepithelial electrical resistance measurement

Transepithelial electrical resistance (TEER) values of iPSc-derived intestine cells were measured at the beginning and end of the coculture using Millicell ERS-2 (Merck Millipore, USA). Measurements were performed in a clean bench using the supplied electrode according to the manufacturer's instructions. Resistance readings from the measurement device were recorded and then TEER value was calculated.

### CYP enzyme activity evaluation using liquid chromatography-tandem mass spectrometry

The CYP enzyme activity was performed at the end of coculture period by introducing specific CYP substrates into the cell culture and measuring its corresponding metabolites concentration using liquid chromatography-tandem mass spectrometry (LC-MS/MS). List of substrates and metabolites is given in Table S1. To focus the evaluation on the PXB-cells, culture inserts of iPSc-derived intestine cells were taken out from the coculture wells. The PXB-cells were incubated with the substrate mixture in HBSS (+) for 4 h at 37 °C and 5% CO_2_. Concentration of substrates and metabolites from aliquots collected at 0, 2, and 4 h were measured. The detailed information on the LC-MS/MS measurement is provided in previously published study (8, 14).

### RNA-sequencing

At the end of coculture, RNA was extracted using the FastGene RNA Basic Kit (Nippon Genetics, Japan) according to the manufacturer's protocol. Total RNA was analyzed and quantified using NanoDrop (Thermo Scientific, USA). RNA samples were sequenced at sequencing facility using Illumina NovaSeq 6000 with NEBNext Directional Ultra RNA Library Prep Kit for strand specific library. RNA-sequencing data analysis was performed following common practices (17, 18).

Data analysis was performed using Galaxy (19), an open-source web-based platform which include various modules for RNA-seq. Raw sequencing data read quality was checked using FastQC module, then trimmed to get rid of low-quality reads using Cutadapt module with following settings: “Filter options minimum length: 20” and “Quality cutoff: 20.” The resulting reads were aligned against human reference genome assembly (hg38) using STAR (20) module. Read counting was performed using featureCounts (21) module with reverse-stranded library setting. Analysis of the differential gene expression was performed using edgeR (22) module using the default normalization method. To extract differentially expressed genes (DEGs) due to crosstalk, pairwise comparisons were performed on monocultured and cocultured PXB-cells from all experiment groups. On the other hand, to obtain DEGs due to the effect of perfusion and direct oxygenation, pairwise comparisons were performed on monocultured PXB-cells between the following groups: (i) TCPS to MPS O_2_[–] and (ii) MPS O_2_[–] to MPS O_2_[+], respectively. Fold changes, *P*-value, and Benjamini–Hochberg's false discovery rate (FDR) were reported for each gene. Volcano plots were generated using R. Gene sets with FDR < 0.05 were separated between positive and negative fold changes and then used for enrichments. Gene ontology (GO) term analysis and its visualization were performed using ClueGO (23) in Cytoscape v3.10.0.

### Statistical analysis

Values are presented as mean ± SD from *n*—number of independent experiments, unless stated otherwise. Statistical significance was evaluated by one-way ANOVA followed by Tukey's HSD test for multiple comparisons and by Student’s t test otherwise.

## Results

### Perfusion and increased oxygen supply enhanced PXB-cells functions in coculture

**Fig. 2.**
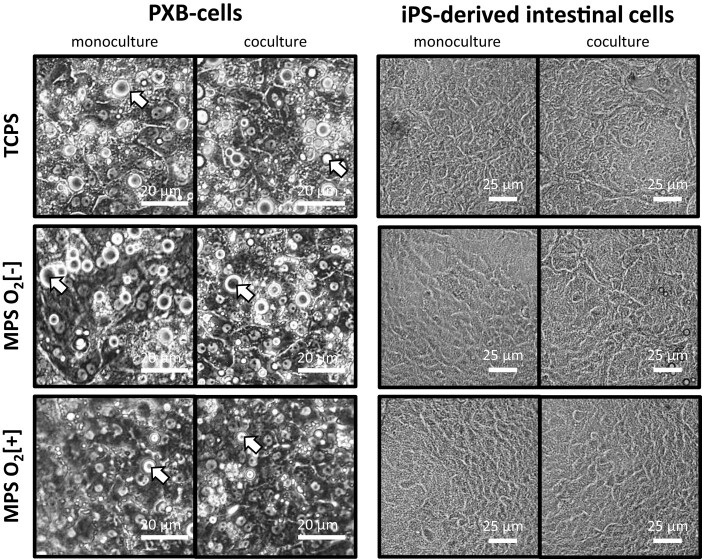
Cell morphology of monocultured PXB-cells and iPSc-derived intestinal cells taken at day 3 of culture by phase-contrast microscopy. Arrow points the lipid droplets in PXB-cells.

The effect of perfusion and direct oxygenation from the MPS toward cell functions were studied by comparison toward conventional cell culture in TCPS plate, which act as control. All experiment groups are shown in Table 1. First, we compared cell morphology taken by phase-contrast microscope at the endpoint of coculture (Fig. 2). The micrographs of PXB-cells and iPSc-derived intestinal cells across all groups showed consistent morphology and confluent monolayers. In the PXB-cells micrographs, lipid droplets can be seen across all conditions (pointed by white arrow). It can be seen that the lipid droplets were significantly less in the MPS O_2_[+] conditions.

**Table 1.** Experiment groups and variables.

Group	Perfusion	Direct oxygenation
TCPS (control)	×	×
MPS O**_2_** [–]	○	×
MPS O**_2_** [+]	○	○

The cells in control group were cultured using standard 24-well type TCPS. In the MPS, perfusion was achieved by using microstirrer pump and direct oxygenation by using oxygen permeable membrane PMP.

**Fig. 3.**
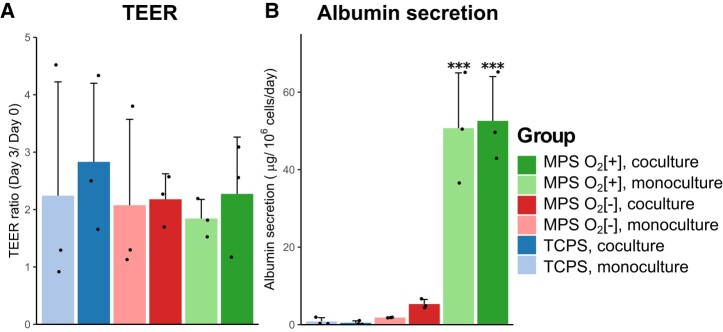
A) TEER value of day 3 compared to day 0 from the iPSc-derived intestinal cells. B) Albumin production of PXB-cells over 3 days of coculture period (***P < 0.001).

Prior to starting coculture, TEER values of the iPSc-derived intestine cells were measured. iPSc-derived intestine cells with TEER values above 100 Ω cm^2^ were considered mature and used in the coculture. TEER measurement was conducted at the beginning (day 0) and endpoint (day 3), which then used to calculate ratio of TEER evolution over the coculture period (Fig. 3A). The average of absolute TEER values at the end of coculture was 467 Ω cm^2^. TEER values across all groups increased roughly 2 times over the coculture period with no significant difference between each group.

We evaluated liver-specific functions by measuring albumin secretion of PXB-cells (Fig. 3B). The albumin secretion from PXB-cells in both MPS was higher than TCPS group. Statistically significant higher albumin secretion value was observed from the MPS O_2_[+] compared to the other two groups. Albumin secretion from cocultured PXB-cells in MPS groups was higher than monocultured one. However, the opposite was observed from the TCPS group where monocultured PXB-cells showed higher albumin secretion.

**Fig. 4.**
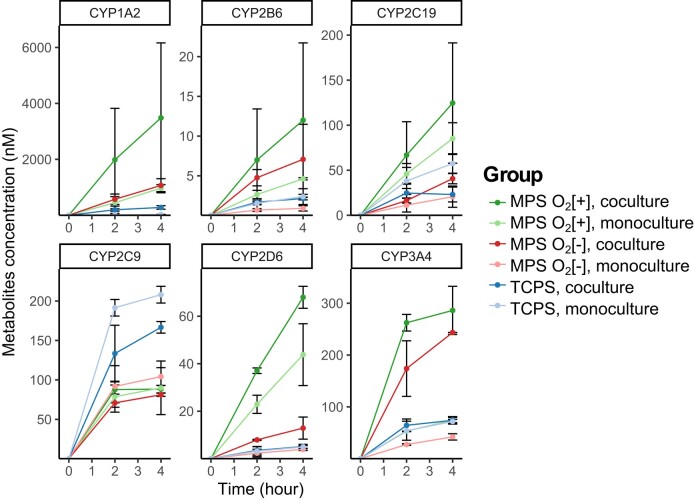
CYP enzyme activity of PXB-cells measured at the endpoint of 3-day coculture period. Values show concentration of specific CYP metabolites in the culture supernatant at certain time point (*n* = 3).

We also evaluated hepatic CYP enzyme activity (Fig. 4) as indicator for drug metabolism by the procedure mentioned in the Methods section. CYP enzyme activities from MPS O_2_[+] group of cocultured PXB-cells were higher compared to the other groups. Within the MPS O_2_[+] group, cocultured PXB-cells also showed higher CYP activity compared to monoculture. Different trend was observed from CYP2C9 where highest CYP activity seems to come from TCPS group. Further investigation by measuring secondary metabolite concentration for CYP2C9 showed that the concentration of CYP2C9 was highest in MPS O_2_[+] group, following similar trend with the other CYPs (Fig. S1).

### ARA as crosstalk factor in the liver–intestine axis

**Fig. 5.**
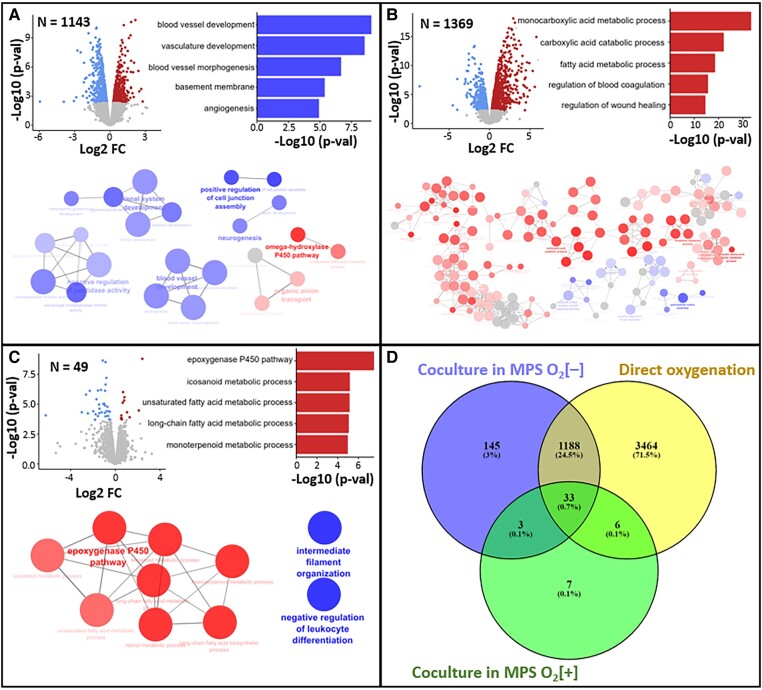
DEG and enrichment results in PXB-cells due to crosstalk across all groups: A) TCPS, B) MPS O_2_[–], C) MPS O_2_[+]. GO terms shown in network to show the distribution of upregulation (red) and downregulation (blue) with top 5 listed in bar chart according to *P*-value. D) Overlap of DEG in PXB-cells due to crosstalk and effect of direct oxygenation (N = number of DEG).

**Fig. 6.**
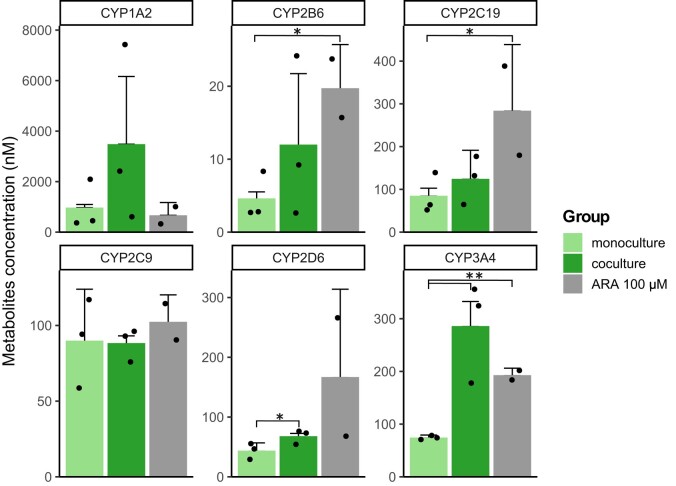
Comparison of CYP activity changes in PXB-cells due to coculture in MPS O_2_[+] condition and addition of ARA in the culture medium (*P < 0.05; **P < 0.01).

The number of DEGs due to coculture varies between groups, with the least found on MPS O_2_[+] (Fig. 5A–C). In the TCPS group, most of the significant GO terms were downregulated. Meanwhile, there were more upregulated GO terms from both of the MPS groups. In the MPS O_2_[+] condition, the GO term with the most significant upregulation is “epoxygenase P450 pathway” which is included under parent GO term of “arachidonic acid metabolic process”. Following this result, we studied whether there is any correlation between ARA and the enhancement of drug metabolism that we observed due to crosstalk. We added ARA into the culture medium of monocultured PXB-cells in MPS O_2_[+] and compared the CYP activity with that from pure monoculture and coculture (Fig. 6). For CYP2C9, coculture and ARA gave similar activity to that from monoculture, which imply no enhancement. For CYP1A2, coculture seemed to increase enzyme activity, while ARA did not. For the rest of studied CYP enzymes, both coculture and ARA seemed to result in enhancement of enzyme activity in different magnitude although mostly not significant (*P* > 0.05).

## Discussion

Several studies (6–8) have demonstrated that coculture of small intestine and liver cells enhanced hepatic drug metabolism, implying the effect of crosstalk between the two organs. However, the mechanism is still unclear, and it is also challenging for in vivo studies to focus on such pure organ crosstalk. On the other hand, there is growing attention in using multiorgan MPS as alternative to animal model in drug development. One of the main advantages of MPS over in vivo studies is that it allows study of specific organ combinations, which should help in understanding systemic behavior of human body. Therefore, in this study, we aim to elucidate such crosstalk between small intestine and liver using highly functional cell sources and provide appropriate MPS features (perfusion and direct oxygenation). We characterized the effect of MPS features on the cell functions to determine best condition to study the crosstalk mechanism. Then, we performed RNA-sequencing to search for potential crosstalk mechanism. Our finding suggests that bile acid may stimulate intestinal lipoprotein release which in turn enhanced hepatic drug metabolism. This demonstrated that MPS with appropriate features and cell sources can be used for studying organ crosstalk study while also furthers our understanding on how crosstalk between gut and liver modulates drug metabolism.

Perfusion and direct oxygenation were considered as the main MPS features because we hypothesized it to be necessary for: (i) enabling transport of signaling molecules responsible for the crosstalk and (ii) maintain high functions of the hepatocytes. To confirm the effect of those MPS features on both PXB and iPSc-derived intestine cells, we compared cell functions when cultured in MPS to the conventional culture method. We confirmed that cell morphology was consistent across all conditions, which indicates that there was no negative impact from perfusion and direct oxygenation toward the cells at microscopic level (Fig. 2). Lipid molecule droplets were observed in the micrographs of PXB-cells (shown by white arrows). In the PXB-cells, implanted human hepatocytes cannot respond to the growth hormones of mice, thus making them less effective in metabolizing lipids, resulting in accumulation of lipid droplets. Previous study has shown that hyperoxic culture of PXB-cells reduce the accumulation of such lipid droplets. This agrees with what we observed, where MPS O_2_[+] conditions having less lipid droplets (24). At the functional level, we studied TEER and albumin secretion. TEER is a good indicator as it informs us the quality of the cell monolayer which is an important function for intestinal epithelial cells as absorption site (25). On the other hand, albumin is a common biomarker used to assess the liver function of cultured hepatocytes (26). According to our results, there was no difference in TEER values due to perfusion and direct oxygenation (Fig. 3A). This is within expectation because the iPSc-derived intestine was placed on cell culture insert placed at certain height above the bottom of MPS device (Fig. 1) where the oxygen permeable membrane and perfusion microchannel are located. Therefore, the iPSc-derived intestine cells only exposed to gentle perfusion and oxygenation effect. Regardless, the TEER values were maintained at similar level compared to the control conditions, which means the function of iPSc-derived intestine cells was not compromised due to the additional MPS features.

Direct oxygenation and perfusion from the MPS greatly enhanced PXB-cells albumin secretion (Fig. 3B), although this is probably overestimated seeing the value in our control condition was lower than the known value from other study (9). This is most likely because in our control condition, we used 1 mL culture medium per well, instead of the commonly used 0.5 mL per well. To ensure smooth perfusion between the compartment, 1 mL culture medium was used in the MPS. Thus, for the sake of consistency, we also used 1 mL on the control condition. Despite this, the albumin secretion value from MPS O_2_[+] was within the range of estimated albumin production in vivo (26). Our result also agree with previous study that reported function and metabolic activity of primary hepatocytes becomes closer to in vivo when it is cultured in oxygen-rich condition (27).

With appropriate conditions from direct oxygenation and perfusion, crosstalk seemed to enhance hepatic drug metabolism. This can be seen from most of the CYP enzyme activity, the highest values were from cocultured PXB-cells in MPS O_2_[+] (Fig. 4). In order to see the relevancy of these CYP activities with that in human body, we compared the rate of metabolites formation calculated from human plasma concentration data in various studies (28–31). The comparison showed that overall the rate of metabolite formation in MPS is lower than that in human body. For CYP1A2 and CYP2B6, the rate is about 1,000 times lower, while for CYP2C9 and CYP3A4 it is about 10 times lower. However, the plasma concentration value for CYP3A4 is within range when compared to that found in mice (32). Although quantitatively the CYP activity is lower than that of the human body, it is still relevant in vivo. Furthermore, these results qualitatively showed that direct oxygenation, perfusion, and crosstalk enhanced hepatic drug metabolism.

One notable finding in our result was from CYP2C9, where the highest activity found in control group. Diclofenac was used as the CYP2C9 enzyme activity test substrate, which can be metabolized through hydroxylation to form hydroxy diclofenac (DF-OH) and conjugated to form acyl-glucuronide diclofenac (DF-AG). Focusing on the 4-h time point, we found that formation of DF-AG was preferred in the MPS group compared to the control (Fig. S1). Considering that DF-AG was reported as the major metabolite form found in the plasma of clinical studies (33), this result implies that under perfusion and direct oxygenation from the MPS, the function of PXB-cells is sufficiently high to favor secondary metabolism, similar to in vivo.

Further results from RNA-sequencing also confirmed the positive effect of perfusion and direct oxygenation for observing in vitro crosstalk (Fig. 5A–C). In the TCPS group, most of the affected GO terms were downregulated, such as “angiogenesis” and “basement membrane”. Meanwhile, from both of the MPS groups, most of the affected GO terms were upregulated, with the top-5 GO terms related to fatty acid metabolic process. We believe this indicates that conventional coculture method in TCPS plate resulted in negative effect to the PXB-cells. On the other hand, it confirmed that perfusion and direct oxygenation supported the coculture.

While both MPS O_2_[+] and MPS O_2_[–] group showed common upregulation on GO terms related to fatty acid metabolic process, there is marked difference in the number of DEGs. We suspect that direct oxygenation greatly affected both monocultured and cocultured PXB-cells in the MPS O_2_[+] group, therefore resulting in less DEGs. This is supported by looking at overlap of DEGs purely due to the effect of direct oxygenation and coculture on both MPS groups (Fig. 5D). We believe that the DEGs that remained even after the effect of direct oxygenation means it plays an important role in the crosstalk mechanism. Thus, we selected the MPS O_2_[+] group as appropriate starting point for further investigation.

The most significant upregulated GO term in the MPS O_2_[+] group was found to be related to ARA, which is a kind of polyunsaturated fatty acid found in the phospholipid membrane of mammalian cells (34). The various parts of human body such as brain, muscle, and liver phospholipid fatty acid constituted up to 25% ARA (35). Known physiological roles of ARA in human body include control of cell membrane fluidity, cell deaths and signaling molecules (especially known from its metabolites) (36). This upregulation suggest that ARA may play important role in the crosstalk between the two cells. It is worth noting as well, preliminary proteomics result from our group's previous study (8) indicated that there was significantly higher lipoprotein secreted from intestinal cells which might increase CYP gene expression in hepatocytes. Such intestinal lipoprotein has been reported to be secreted in response to the presence of bile acids (37). As a polyunsaturated fatty acid, there is possibility that ARA could be one of the constituent for intestinal lipoprotein.

**Fig. 7.**
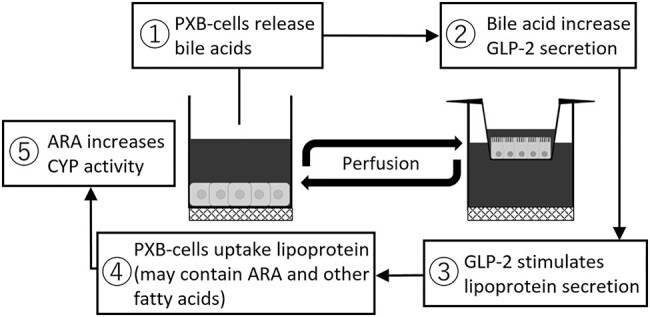
Diagram of the speculated liver–intestine crosstalk mechanism which results in modulation of drug metabolism.

From these pieces of information, we hypothesize some form of stimulus such as bile acid from the PXB-cells lead to iPSc-derived intestine cells releasing lipoprotein (that include ARA as its component) which then transported into the PXB-cells and enhanced CYP activity (Fig. 7). There are several points that we observed which supports this hypothesis: (i) Gene expression of BSEP and CYP7A1 (Fig. S2), the major transporter for bile acids secretion and synthesize, was high for both monoculture and coculture of PXB-cells. (ii) Gene expression for GLP2R (Fig. S2), receptor of glucagon-like-peptide-2 (GLP2), was significantly higher in coculture than monoculture of iPS-derived intestine cells. GLP2 has been reported to be stimulated by bile acids (38) and in turn increases intestinal lipoprotein secretion (39). (iii) Addition of ARA to monoculture of PXB-cells significantly enhanced the activity of several CYPs with general tendencies where coculture and ARA are higher than that of monoculture (Fig. 6).

It is still unclear how PXB-cells uptake ARA and initiates cascade that resulted in enhancement of CYP activity in our culture system. However, the involvement between ARA and drug metabolism, specifically the induction of CYP enzymes has been mentioned in several studies. ARA has been known for its ability to be metabolized through cyclooxygenases, lipoxygenases, and CYP epoxygenases (CYP2C, CYP2J) to form various active fatty acid mediators (34). Regulation of CYPs could be generally attributed at the transcriptional level by aryl hydrocarbon receptor (AhR), constitutive androstane receptor, and pregnane X receptor (40, 41). Eicosanoids, one of the common forms of ARA metabolite, was reported as a ligand for AhR. Another ARA metabolite, prostaglandin, was also reported to be endogenous substrate of some CYPs.

Although the presented crosstalk mechanism remains speculative, we believe that the current evidences are useful as starting point for further studies and also prove the usefulness of the culture system using MPS and appropriate features to elucidate organ crosstalk in vitro. In order to further prove the current speculated crosstalk mechanism, future studies measuring bile acids, GLP2, and lipoprotein (ARA) in the system are promising.

In this study, as we focused on drug metabolism, we did not include nonparenchymal cells, which might play an important role in crosstalk for diseased state through cytokines and other kind of signaling molecules. Incorporating nonparenchymal cells through organoids or hierarchical coculture might be an avenue for further study investigating more complex crosstalk. Deeper study that incorporates multiomics approaches on all the involved cell types and global change in metabolites, protein secretions, genes, and transcription factors may help to elucidate crosstalk mechanism in much more detail. Nevertheless, here we have demonstrated that simple MPS with the suitable features and cell sources could be used to investigate and gain new insight on unknown multiorgan crosstalk.

## Conclusion

In this study, we speculated the potential gut–liver crosstalk mechanism where bile acids from the liver stimulate the secretion of intestinal lipoprotein containing ARA, which is then taken up by liver to enhance hepatic drug metabolism. We confirmed that perfusion and direct oxygenation were necessary to maintain cell functions which ultimately allowed us to study the pure crosstalk between the two organs. Overall, our study demonstrated that MPS with appropriate features and cell sources can be used to study novel crosstalk between any specific combination of organs which is challenging to do using in vivo approaches.

## Supplementary Material

pgae070_Supplementary_Data

## Data Availability

The RNA-seq data used in this study can be accessed in the Gene Expression Omnibus repository with the accession number GSE244407. Other information for raw data and codes are available upon reasonable requests to the corresponding author.
