# Supplementary material for: Gut–liver microphysiological systems revealed potential crosstalk mechanism modulating drug metabolism
Source: PNAS Nexus. 2024 Feb 9;3(2):pgae070. doi: 10.1093/pnasnexus/pgae070 (PMC10879850; doi:10.1093/pnasnexus/pgae070)
Supplement: pgae070_Supplementary_Data [file pgae070_supplementary_data.docx]

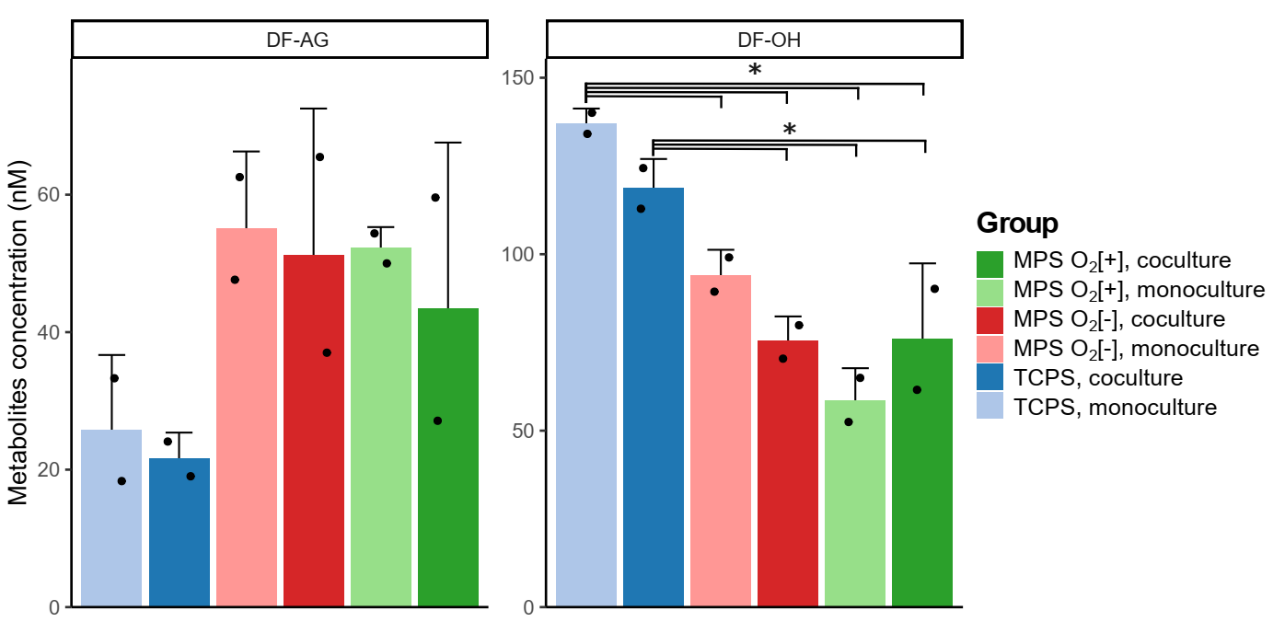


**Figure S1.** Activity of CYP2C9 enzyme observed through concentration of its primary (hydroxy diclofenac) and secondary metabolite (diclofenac acyl glucuronide). (*p < 0.05)


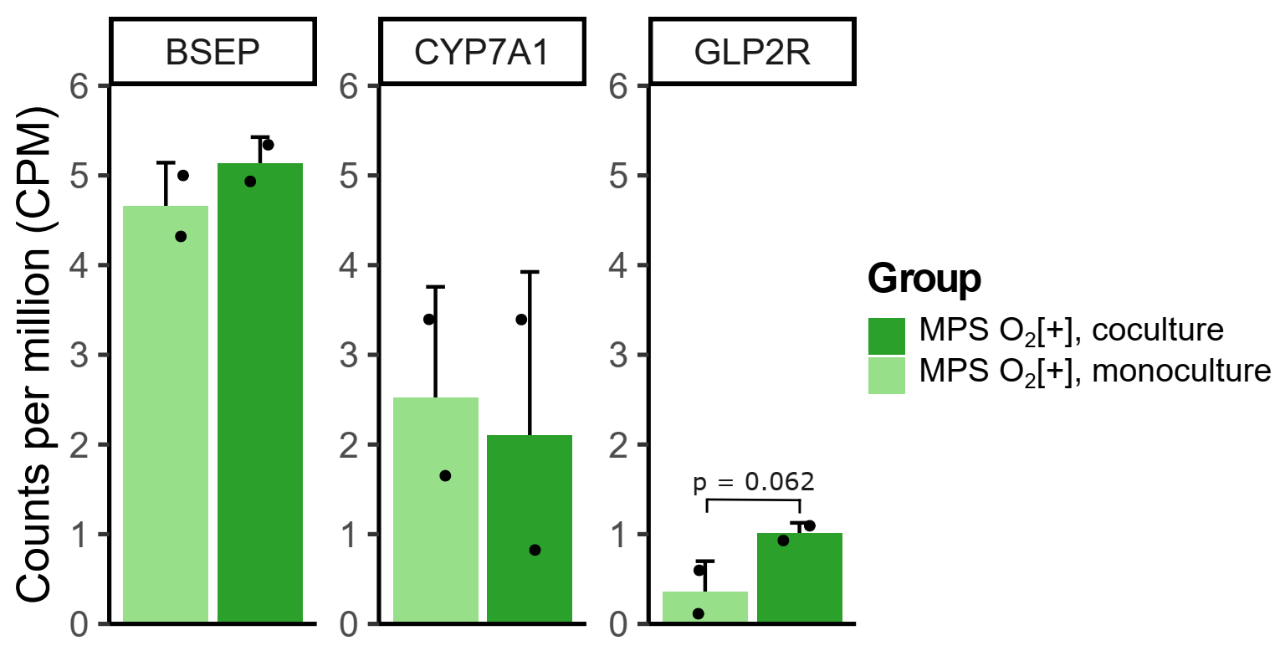


**Figure S2.** Gene expression for BSEP and CYP7A1 in PXB-cells and GLP-2 receptor in iPSc-derived intestine cells from the MPS O_2_ [+] condition.

| Target | Substrate | Substrate concentration in mixture (µM) | Metabolite |
| --- | --- | --- | --- |
| CYP1A2 | Phenacetin | 20 | Acetaminophen |
| CYP2B6 | Bupropion | 5 | OH-Bupropion |
| CYP2C9 | Diclofenac | 1 | 4-OH Diclofenac,  Diclofenac acyl glucuronide |
| CYP2C19 | Mephenytoin | 40 | 4-OH Mephenytoin |
| CYP2D6 | Bufuralol | 5 | 1-OH Bufuralol |
| CYP3A4 | Midazolam | 2 | 1-OH Midazolam |

**Table S1.** List of substrates and metabolites used for CYP enzyme activity analysis.
